# Reference-Free Vibration-Based Damage Identification Techniques for Bridge Structural Health Monitoring—A Critical Review and Perspective

**DOI:** 10.3390/s24030876

**Published:** 2024-01-29

**Authors:** Mohammad Moravvej, Mamdouh El-Badry

**Affiliations:** 1 T.Y. Lin International Canada Inc., Vancouver, BC V6C 1S4, Canada; 2 Department of Civil Engineering, University of Calgary, Calgary, AB T2N 1N4, Canada

**Keywords:** bridges, damage identification techniques (DITs), reference-free, structural health monitoring (SHM), vibration

## Abstract

Bridges are designed and built to be safe against failure and perform satisfactorily over their service life. Bridge structural health monitoring (BSHM) systems are therefore essential to ensure the safety and serviceability of such critical transportation infrastructure. Identification of structural damage at the earliest time possible is a major goal of BSHM processes. Among many developed damage identification techniques (DITs), vibration-based techniques have shown great potential to be implemented in BSHM systems. In a vibration-based DIT, the response of a bridge is measured and analyzed in either time or space domain for the purpose of detecting damage-induced changes in the extracted dynamic properties of the bridge. This approach usually requires a comparison between two structural states of the bridge—the current state and a reference (intact/undamaged) state. In most in-situ cases, however, data on the bridge structural response in the reference state are not available. Therefore, researchers have been recently working on the development of DITs that eliminate the need for a prior knowledge of the reference state. This paper thoroughly explains why and how the reference state can be excluded from the damage identification process. It then reviews the state-of-the-art reference-free vibration-based DITs and summarizes their merits and shortcomings to give guidance on their applicability to BSHM systems. Finally, some recommendations are given for further research.

## 1. Introduction

Bridges are integral components of transportation infrastructure and play a vital role in connecting communities and facilitating economic activities. However, over recent decades, the integrity of many bridges worldwide has been compromised due to a combination of factors such as aging, insufficient maintenance, adverse environmental conditions, excessive loading, and unforeseen extreme events like earthquakes, hurricanes, and floods. Moreover, the burgeoning urban population and expanding economy have placed increasing demands on aging bridges, raising global concern about their structural performance and safety against catastrophic failures [1,2]. The tragic collapse of the I-35W Mississippi River Bridge near Minneapolis in 2007, just two years after being classified as structurally deficient, emphasizes the dire consequences of operating severely deteriorated bridges [3,4]. This incident, along with other notable bridge failures [5], underscores the critical importance of continuously assessing the structural health of bridges to ensure a safe and efficient operation throughout their service life. In pursuit of this objective, various national codes and standards have been developed around the world with diverse methodologies for condition assessment and inspection of existing bridges. However, more specific technical codes are still required to provide more detailed guidelines in practice to maintain structural safety of bridges [6,7].

To address this imperative, the early detection of distress in bridges is paramount, necessitating the utilization of Damage Identification Techniques (DITs). In this context, damage encompasses adverse changes induced in the materials, physical and mechanical properties, and interconnections of bridge components that compromise their present or future performance [8]. A robust DIT can detect the presence of damage, determine its location, and quantify its severity [9]. Furthermore, the outcome of a successful DIT can be used for prediction of the remaining service life of bridges. Therefore, damage identification assumes a central role in Bridge Structural Health Monitoring (BSHM) systems. An effective BSHM system can detect any distress, identify potential and impending failures, and notify bridge owners to halt operations before any catastrophic failure occurs, thus leading to an expedited decision-making process regarding maintenance and/or repair [8]. Integrating digital twin models into bridge maintenance systems enhances their effectiveness by facilitating a comprehensive and data-driven approach. This involves continuous updating of a digital replica of the bridge with measured data and DIT results, providing an accurate reflection of its current state [10].

Over recent decades, the field of BSHM has witnessed considerable attention from researchers and bridge owners, leading to the development of a wide variety of damage identification techniques, among which vibration-based DITs have shown great potential [2,11]. Vibration-based DITs consider the bridge response as a function of its physical attributes (e.g., stiffness, mass, and damping) of the structural elements and their connections. They rely on vibration measurements to discern damage-induced effects in the bridge response and its extracted dynamic parameters, such as natural frequencies, mode shapes, etc.

Vibration-based DITs have been extensively investigated over the past few decades. One of the earliest comprehensive reviews of these techniques and their applications in Structural Health Monitoring (SHM) was conducted by Doebling et al. [11,12], primarily focusing on practical considerations. Subsequently, Sohn et al. [13] presented an updated review, emphasizing the statistical pattern recognition paradigm in SHM. Fan and Qiao [14] presented a comprehensive review of modal parameter-based DITs including techniques based on natural frequency, mode shapes, mode shape curvature, and hybrid approaches that combine mode shapes and frequency information. The review concluded that mode shape-based and mode shape curvature-based techniques, when coupled with optimization methods and signal processing tools, could accurately pinpoint damage locations. A review on the development of modern signal processing tools, such as wavelet transform, and artificial intelligence optimization methods, such as genetic and neural network algorithms can be found in [15]. Das et al. [16] conducted a comparative study on non-modal-based methods versus modal-based methods. They concluded that the time series methods might be the most effective vibration-based DITs. Casas and Moughty [17] reviewed recent developments in modal and non-modal vibration-based DITs for short and medium span bridges. They noted challenges associated with the implementation of modal-based techniques in existing bridges due to difficulties in the extraction of modal properties under ambient excitations.

While the aforementioned literature reviews focused primarily on techniques that require prior knowledge of the dynamic response of the structures in their undamaged state to be used as reference baseline data for damage identification, it is important to acknowledge that such response data are often unavailable in in-situ scenarios. Consequently, researchers have recently dedicated efforts to developing reference-free DITs that circumvent the need for a reference state. This paper aims to comprehensively review the state-of-the-art in reference-free vibration-based DITs applicable to BSHM systems.

In the subsequent sections, we elucidate the concept of the reference state within the damage identification process and outline the limitations imposed on the vibration-based DITs that rely on this state. We then delve into how reference-free techniques overcome these limitations by obviating the need for the reference state. Avoiding unnecessary technical complexities, this paper provides a straightforward explanation of the development process of reference-free techniques and assesses their strengths and weaknesses. Finally, some recommendations are offered for further research and development in the realm of reference-free vibration-based damage identification in bridges.

## 2. The Reference State in Vibration-Based Damage Identification

In reference-based DITs, damage is typically defined as undesirable *changes* induced in the material and physical properties that influence performance of bridge structural systems [8]. Detecting these changes involves comparing the dynamic properties of the bridge in two distinct structural states: the current state and the reference state. The current state represents the present structural condition of the bridge, suspected of being affected by damage. The damage identification process aims to detect the presence of damage, determine its location, and estimate its severity in this current state. In contrast, the reference state denotes the initial, undamaged condition of the bridge. It is also referred to in the literature as the *undamaged*, *healthy*, *intact*, and *pristine* state [18,19,20,21,22].

In an early definition of the reference state, Stubbs and Kim [22] suggested that the reference structure be realized as a structure with the same topology as the current structure, excluding any damage accumulated over time. In the studies conducted on vibration-based DITs, the reference state is often referred to as the *baseline state* because the dynamic response measured in this state serves as the baseline data—sometimes referred to as the *footprint* [21]—in the damage detection process. All subsequent measurements are then compared to these baseline data to identify damage-induced changes in response. Hence, these DITs heavily depend on prior knowledge of the reference state.

However, the reliance on baseline data poses a significant limitation for reference-based techniques [20] because in many real-world scenarios, particularly in in-situ cases, obtaining structural properties of the pristine structure can be challenging or even impossible [14,22,23]. This challenge arises because most damaged bridges were constructed several decades ago, and no dynamic response data were recorded in their undamaged state at the time [21]. Consequently, there are often no baseline data available to represent the reference state of bridges before they were exposed to various sources of damage.

Even if the response of a newly constructed bridge has been recorded since its early stages of operation, it cannot be assumed with absolute certainty that this response was unaffected by unknown defects in the bridge or its monitoring system. Damage can exist in varying degrees in all engineered systems [8]. Therefore, if a bridge appears to continue functioning as designed, it cannot be assumed to be entirely free of damage. In essence, an actual reference state representing an entirely undamaged bridge condition either does not exist or is unattainable in most in-situ cases. Consequently, reference-based DITs often resort to assuming the very first available state of the bridge as the reference state, from which the necessary baseline data are derived. However, making such an assumption, especially when the initial state of the bridge may be slightly or severely affected by unidentified damage, can compromise the effectiveness and reliability of the damage detection process.

Additionally, bridges are subject to varying operational and environmental conditions, including changes in traffic loads, temperature, humidity, and more. These variations can induce significant changes in the dynamic response of bridges [24]. These changes in response due to fluctuating conditions can be mistakenly interpreted as damage-induced changes, leading to false-positive damage indications. Halting or disrupting normal bridge operation due to false damage indications can result in substantial costs for bridge owners and negatively impact their trust in the BSHM systems. Furthermore, variations in measured dynamic responses due to changing operational and environmental conditions may mask actual damage-induced changes in the response. In some cases, the changes due to varying conditions may even exceed those caused by structural damage [24]. For example, Ko and Ni [25] discussed the impact of temperature on highway bridge modal variability, revealing that changes in natural frequencies could reach up to 10% due to temperature variations, potentially exceeding changes caused by structural damage. Another illustration comes from nine-month long tests conducted by Alampalli [26,27] on a steel-stringer bridge with a concrete deck, where the changes in natural frequencies caused by freezing of the bridge supports were in order of magnitude larger than those due to intentional saw cuts across the bottom flanges of the bridge girders. Several other studies discussed by Zhou and Yi [24] and Ko and Ni [25], have illustrated the sensitivity of bridge dynamic responses to operational and environmental conditions. This sensitivity can lead to false-negative damage indications, where no damage is indicated even when it actually exists.

As summarized in Table 1, damage identification based on comparisons of the dynamic response of bridges measured at the current state with the one obtained at the reference state can be significantly inefficient for two primary reasons. First, the response data for the reference state are often unavailable in most in-situ cases and they must be assumed with a high level of uncertainty. Second, comparisons between responses obtained from the current and assumed reference states can be substantially affected by the variable operational and environmental conditions. As a result, the development of DITs that permit implementation of SHM systems in bridges without the need for prior knowledge of a reference state has become increasingly important [28].

Recent research efforts have focused on the development of vibration-based damage identification techniques that rely solely on the dynamic response obtained from the current state of bridges, without requiring baseline data from a reference state. These techniques are referred to herein as *reference-free* vibration-based damage identification techniques. However, it is essential to note that reference-free techniques are intended to eliminate the need for a reference state but still incorporate some implicit knowledge of a baseline, such as the smoothness of mode shapes or linear characteristics of response, to represent the expected structural behavior of an undamaged bridge in its initial state, as will be further discussed later in this paper.

## 3. Reference-Free Vibration-Based DITs

Reference-free techniques are based on the premise that the response obtained from the current structural state of a bridge provides sufficient data for the damage identification process. This response typically pertains to either the time or space domain of the bridge behavior. In the time domain, the response captures the temporal variation of a specific parameter such as the acceleration signals of the bridge vibration at various locations. The time domain-based techniques leverage advanced signal processing tools to analyze the measured response, unveiling hidden characteristics that may serve as indicators of damage. Conversely, in the space domain, the response reveals details about the bridge deformation along its span. This includes information related to mode shapes and mode shape curvature, which are derivatives of the spatial response. The space domain-based techniques have gained considerable attention in structural damage identification due to their straightforward interpretability, as deformations are directly linked to the structure’s physical properties. However, it is worth noting that a transformation is often required to derive the space-domain response from the measured time-domain response [2]. The conventional methods for assessing a structure’s response involve gathering vibration data, such as deck vertical acceleration, at various time intervals. Subsequently, the collected data undergo analyses, such as modal analysis, to unveil spatial information like deck mode shapes. Further details on this process are provided in Section 5.

The following sections describe the core components of reference-free vibration-based DITs and provide a review of the latest research in this field, categorizing the approaches into either time or space domain-based methodologies.

## 4. Reference-Free Time Domain-Based Techniques

### 4.1. Fundamental Steps

In general, there are three fundamental steps in the time domain-based reference-free damage identification process. The initial step involves capturing the bridge response in the time domain amidst dynamic excitations. Subsequently, the second step focuses on identifying the effects of structural damage within the recorded response. The third step involves quantifying the detected damage-induced effects, allowing the techniques to distinguish between the damaged and undamaged areas. Notably, certain time domain-based techniques possess the capacity to gauge severity of the detected damage without the need for any data from a reference state.

**Figure 1:**
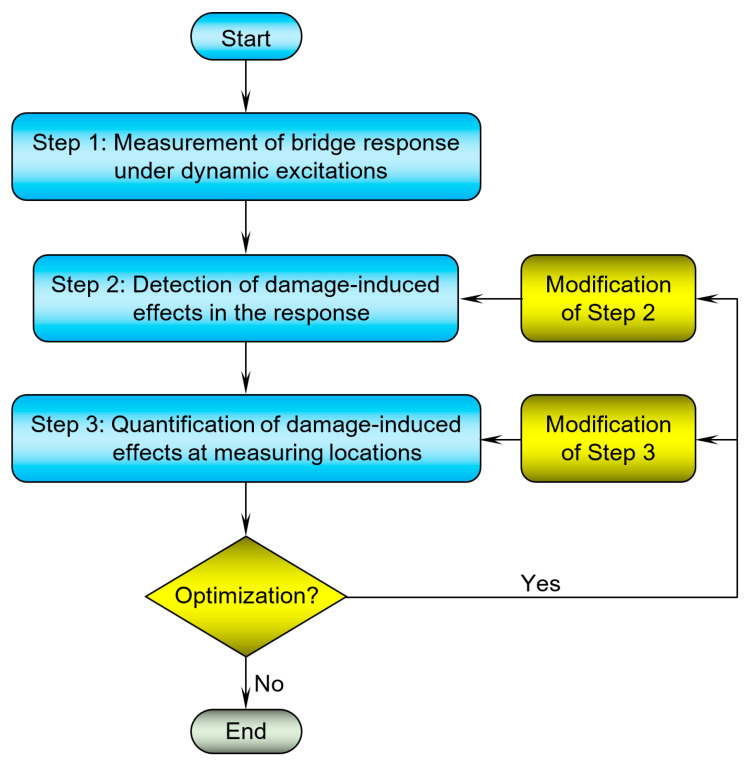
Flowchart of time-domain DITs.

Some advanced time domain-based techniques further optimize the damage identification process through integration of artificial intelligence optimization techniques, such as genetic and neural network algorithms, to enhance the efficiency of damage identification results by maximizing the distinguished differences in the extracted characteristics of responses obtained from the damaged and undamaged locations. This enhancement can be achieved through modifications in the second and/or the third main steps as illustrated in the flow chart of Figure 1.

The initial step plays a pivotal role in practicality of the time domain-based techniques. In addition to the type and number of sensors required for response measurement, the sensor placement is crucial for efficient and straightforward data collection. Some techniques require simultaneous measurements from all designated locations under the same dynamic excitation, while others allow for intermittent measurements using a limited number of sensors relocated from one set of locations to another. The choice of source of dynamic excitations is also a significant practical factor, favoring techniques that rely on ambient sources such as wind and traffic loads over those necessitating bridge operation interruption for dynamic excitation tests [29]. Utilizing shock and vibration testing shakers, typically used for exciting small-scale lab specimens in a broad range of frequencies, proves infeasible for large and heavy structures such as bridges.

**Figure 2:**
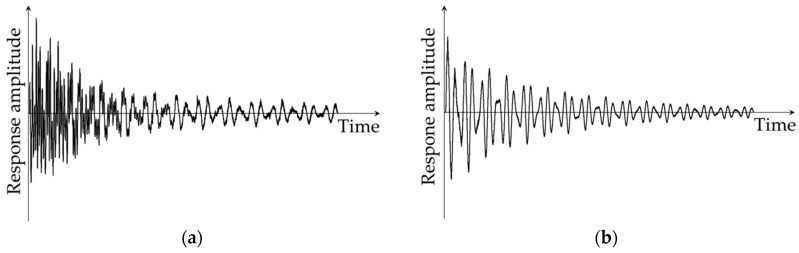
Damage-induced disturbances in (**a**) response affected by severe damage compared with (**b**) response affected by slight damage.

In the second step of the damage identification process, modern signal processing tools are often utilized to detect the damage-induced effects within the measured dynamic responses. Compared to responses obtained from undamaged locations, responses influenced by damage usually contain greater disturbances. Figure 2 schematically illustrates the effects of damage on the amplitude of a time domain response.

Among advanced signal processing tools, the wavelet transform (WT) has shown great potential for detecting localized damage-induced effects in the response of bridges [30]. The WT is a powerful signal processing tool and mathematical converter that can disclose the hidden characteristics of signals [31]. In the application of time domain-based DITs, the hidden characteristics are the damage-induced effects in the measured dynamic response. Generally, WT can take a continuous form, known as *continuous wavelet transform* (CWT), or a discrete form, known as *discrete wavelet transform* (DWT).

**Figure 3:**
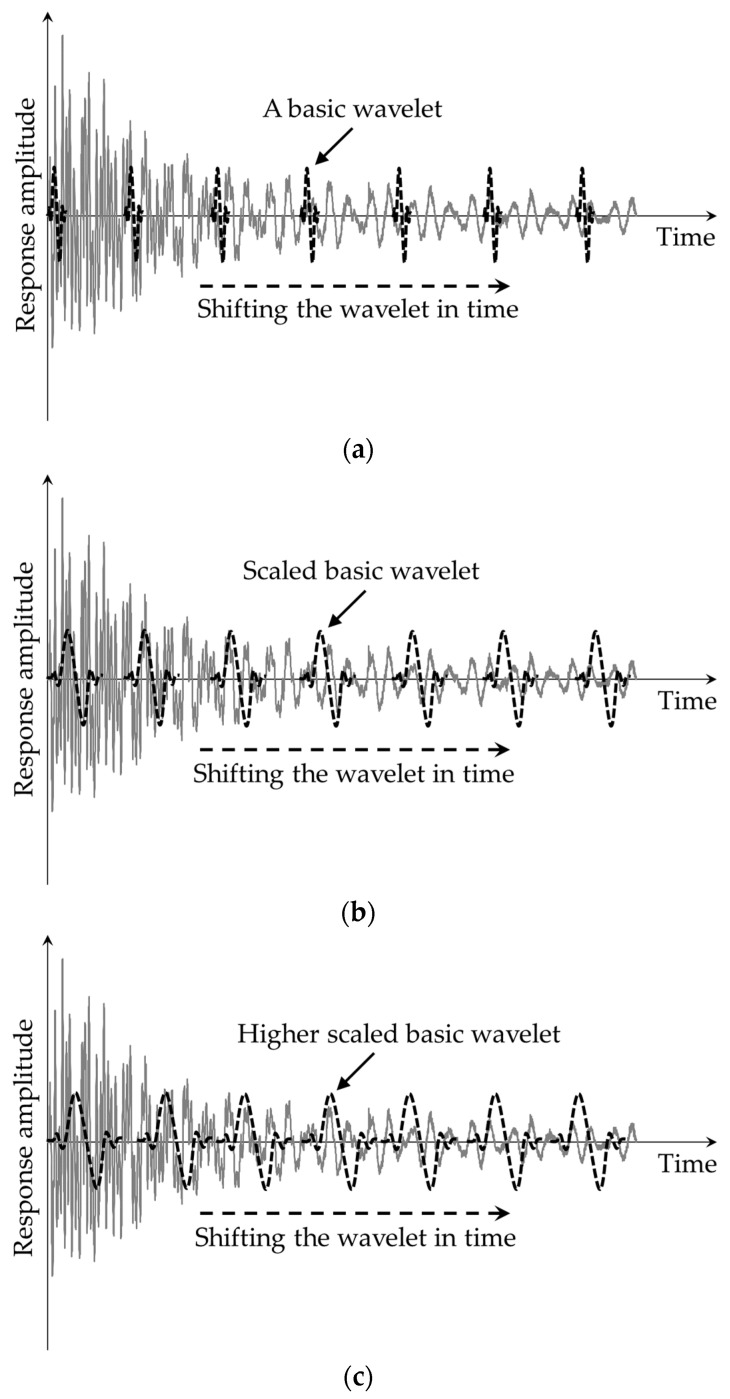
The process of shifting and scaling a basic wavelet function in the CWT of a response: (**a**) Shifting a basic wavelet in time; (**b**) Shifting a scaled (low frequency) version of the basic wavelet in time; (**c**) Shifting a higher scaled (lower frequency) version of the basic wavelet in time.

The CWT evaluates the correlation between a basic wavelet function, ψ(t), and a continuous signal, a(t), such as a continuously measured acceleration response from bridge vibrations. The basic wavelet can be seen as a small wave that resembles the damage-induced disturbances in the acceleration response. In contrast to Fourier transform, which breaks down a signal into sine waves of varied frequencies and phases, the CWT decomposes the signal by scaling and shifting the basic wavelet function, as illustrated in Figure 3. Consequently, the content of damage-induced disturbances in the response can be calculated using wavelet coefficients, defined by Equation (1), representing the correlation level between the response and the basic wavelet.

(1) $$C\left(s,\tau \right)=\frac{1}{\sqrt{s}}\int_{-\infty }^{+\infty }a\left(t\right)\psi^{*}\left(\frac{t-\tau }{s}\right)dt,$$

where $\psi^{*}\left(t\right)$ is the complex conjugate of the basic wavelet function, shifted by a factor τ and scaled by a factor s.

The DWT is more frequently used in practice as it has shown superior performance over the CWT in detecting damage-induced disturbances in the dynamic response of structures [32]. In the DWT, the dynamic response is sampled at discrete time intervals, Δt, through data acquisition systems, resulting in discretized vibration responses like acceleration response, $A\left(t_{i}\right)$; where $t_{i}=i\;\Delta t$, with i being the time interval number. By adopting the values $2^{j}k$ for the shifting factor and $2^{j}$ for the scaling factor, with j and k being integers, the discrete basic wavelet, $\psi_{j,k}\left(t_{i}\right)$, can be obtained from Equation (2) and the corresponding discrete wavelet coefficients, $C_{j}\left(k\right)$, can be calculated by Equation (3).

(2) $$\psi_{j,k}\left(t_{i}\right)=2^{-\frac{j}{2}}\psi \left(2^{-j}t_{i}-k\right),$$

(3) $$C_{j}\left(k\right)=2^{-\frac{j}{2}}\sum_{i}A\left(t_{i}\right)\psi \left(2^{-j}t_{i}-k\right).$$

The DWT works as a pair of filters decomposing the response into low- and high-frequency components and calculates the corresponding wavelet coefficients for each component. The wavelet coefficients corresponding to the low- and high-frequency components are also called *approximation* and *detail coefficients*, respectively. The approximation coefficients are filtered further into low- and high-frequency components in each subsequent decomposition level up to the last level. The detail coefficients, on the other hand, do not go through filtration beyond the first decomposition level. Thus, in the final level of decomposition, the original response becomes decomposed into groups of wavelet coefficients, from the highest frequency component (i.e., the detail coefficients of the first decomposition level) to the lowest frequency component (i.e., the approximation coefficients of the last decomposition level).

**Figure 4:**
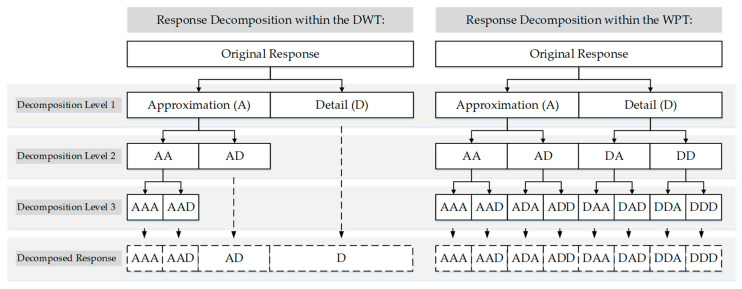
Comparison between the DWT and the WPT for three levels of decomposition [31].

The lack of filtration in the detail coefficients of the DWT results in relatively low resolution, especially in the high-frequency region, posing challenges in disclosing the hidden characteristics of the response. To surmount these challenges, researchers have turned to another discrete wavelet transform, known as *wavelet packet transform* (WPT) [31,33,34,35,36]. Both the DWT and WPT decompose a given signal into low- and high-frequency components. However, while the DWT repeatedly decomposes only the low-frequency component, the WPT decomposes both the low- and high-frequency components at each level of decomposition. Consequently, the WPT provides richer information on high-frequency components by breaking them down into smaller packets leading to higher resolutions. Figure 4 visually illustrates the comparison between the DWT and WPT across three levels of decomposition.

After extracting the hidden characteristics of the time domain response through signal processing efforts, the third step in the damage identification process is focused on quantifying damaged-induced dissimilarities in the responses obtained at different locations. This quantification is used to locate the damage and sometimes estimate its severity. Traditionally, time domain-based techniques utilize the probability theory and statistical analysis methods to quantify any dissimilarities in the response that can indicate the presence of damage.

Contrary to reference-based techniques that compare the responses from the same location but in two different states (current and reference state), reference-free techniques quantify damage-induced dissimilarities by comparing responses from various locations in the same current state. Recently, entropy analysis has found applications in the time domain-based techniques for similar purposes. Generally, entropy is a quantitative measure of the degree of disorder in a system [37]. By analyzing a corresponding distribution of the system, denoted as {*P*}, entropy can be expressed by Equation (4). The distribution may take a form of probabilities, a vector of energy contents, etc., with multiple elements denoted as $p_{1},\;p_{2},\;.\;.\;.,\;p_{M}$ ensuring that the summation of the elements equal one.

(4) $$E\left(\left\{P\right\}\right)=-\sum_{m=1}^{M}p_{m}\;\ell n\left(p_{m}\right).$$

Entropy analysis can be used in combination with wavelet analysis to quantify the degree of damage-induced disorder in the response [38,39]. The combination of wavelet and entropy analyses is often referred to in the literature as *wavelet entropy* (WE). The premise is that an occurrence of structural damage induces local disturbances and increases the degree of disorder in the responses obtained at the damaged locations compared to those obtained at undamaged locations. Thus, the wavelet entropy analysis can detect and quantify damaged-induced disturbances, i.e., dissimilarities imposed by damage in the bridge response.

### 4.2. Review of Reference-Free Time Domain-Based Techniques

Among the first research efforts in the time domain approach that demonstrated the feasibility of damage detection in existing structures without the need for data from a reference state is the work by Ren et al. [36] and Ren and Sun [40]. The primary aim was to develop structural damage detection and localization techniques utilizing wavelet packet energy [36] and wavelet entropy [40] analyses. The techniques were validated through laboratory testing of a 1:3 scaled slab-on-girder bridge model with damage in the slab-to-girder connection simulated by loosening some of the shear connectors. The study concluded that if an undamaged location on the bridge was known beforehand, the reference state of the intact structure could be omitted from the damage detection process. This highlighted the potential of the wavelet packet energy-based and proposed wavelet entropy-based techniques for in-situ applications where no reference data for bridges were available. However, the requirement of prior knowledge of the undamaged locations, and the need to simultaneously measure the response at this location along with all other locations under the same dynamic excitation, can be a major drawback in the practical application of the proposed technique.

In the early stages of the development of reference-free time domain-based DITs, Mikami et al. [41] introduced a technique for damage detection in beam-like structures. The technique they proposed obviated the need for baseline data by considering one half of a damaged beam as the reference for the other half. The fundamental premise was that structural damage induced disturbances in the dynamic response recorded near the damaged location. Although such local disturbances might not be apparent in the measured dynamic response, they could often be detected through wavelet transform. Mikami et al. [41] investigated the efficiency of their proposed technique using numerical simulation and experimental data from dynamic excitation tests conducted on a 2.09 m long steel T-section beam. Although the technique successfully detected damage and its location in the simulated and laboratory tested beam, it was applicable only to symmetrical beam-like structures with symmetrical boundary conditions. Furthermore, since one half of the beam was assumed as the reference for the other half, the baseline data measured for the reference half might also be affected by the damage, potentially leading to false-negative indication of damage and its consequences.

Inspired by the potential of reference-free damage detection techniques and advancements in signal processing tools, Lee and Yun [42] devised a technique tailored for real-time BSHM systems. The technique was based on WPT coupled with a likelihood ratio algorithm to generate a damage-sensitive index. The concept of likelihood was first introduced in mathematical statistics by Fisher in 1922 [43] for quantifying the degree of congruence between a probability distribution and samples. Lee and Yun [42] employed likelihood to quantify dissimilarities in the probability distributions of wavelet coefficients between damaged and undamaged locations. The wavelet coefficients were calculated by performing WPT on time domain responses.

Lee [44] explored an alternative damage detection strategy that replaced WPT with CWT and the likelihood analysis with entropy analysis. The performance of these two techniques in damage detection was compared in a three-dimensional steel truss with loose bolts at multiple joints as a type of damage. The truss was dynamically excited using an electrodynamic shaker, and the response was measured by triaxial accelerometers. The study concluded that although both techniques could detect the damage location(s) without the need for a reference state, the significant computational time required for the CWT would favor the practical application of WPT in real-time BSHM systems. Lee et al. [45] studied the wavelet entropy-based technique further by evaluating it against progressive damage states in the same 3D truss. The results indicated that the proposed technique effectively detected both slight and severe levels of bolt loosening. However, it is essential to note that the above-mentioned investigations were limited to the use of a shaker as a means of dynamically exciting the truss. Moreover, both techniques were applied only to a structure with elements having identical geometric and mechanical properties, and only one type of damage, namely, bolt loosening, was considered [45].

Ravanfar et al. [46] and Ravanfar [47] focused their research on damage detection in beam-like structures using a wavelet entropy-based technique. This technique integrated WPT and entropy analysis to identify the location and extent of damage. The efficiency of the technique was evaluated numerically and experimentally in a set of 3-metre long steel beams with single, double, or triple notch cutting as cases of damage. The study aimed to enhance the wavelet entropy analysis by determining the most suitable parameters through maximizing the difference in the indices calculated for the damaged and undamaged locations. Therefore, genetic algorithms were employed to identify the optimal basic wavelet function and number of decomposition levels. Nevertheless, limitations of the experimental implementation of the proposed technique raised concerns about its generality. For example, only one type of damage was considered in one type of structure. Furthermore, the dynamic excitations were induced using a shaker. In addition, the response measurements necessitated simultaneous acquisition at all designated locations, requiring the number of accelerometers to match the total number of measuring locations. This particular shortcoming significantly limits the practical application of the technique in most in-situ scenarios.

To enhance the practicality of wavelet entropy-based techniques, Moravvej et al. [48] proposed a relative technique capable of identifying different types of damage in various types of bridge structure under ambient excitation. A relative spectral entropy analysis was combined with DWT to compare the responses of locations theoretically possessing similar dynamic properties. The proposed technique works based on the premise that structural damage induces dissimilarities in the wavelet energy distributions of response at damaged locations compared to undamaged locations. Thus, quantifying the damage-induced dissimilarities through wavelet entropy could identify the targeted damage.

The proposed technique was tested on small-scale [48,49,50,51,52] and large-scale [53,54,55] bridge specimens affected by various types of structural damage resulting from static and fatigue loading. Cracking of concrete elements, fracture in internal reinforcement, failure of connections, rupture in confinement tubes, and debonding of strengthening laminates were identified in beams, truss girders, and slab-on-truss girder bridges built of conventional and advanced composite materials. For small-scale specimens and elements, dynamic excitations were produced by applying impact loads using a hammer. For large-scale specimens, the excitations were induced by the application of vertical cyclic load simulating ambient vibration of a bridge due to traffic loading. The dynamic responses of the specimens were measured by up to four accelerometers, the location of which was successively changed to cover all designated measuring locations. The results from these studies showcased the efficiency of the technique in detecting damage, identifying its location, and estimating its severity in different scenarios. Additionally, the performance of the technique was evaluated experimentally under varying operational and environmental conditions [51], demonstrating capability to mitigate the undesirable effects of such variations on the damage identification process.

More recently, a time domain-based DIT employing Savitzky–Golay Filter (SGF) was proposed by Kordestani and Zhang [56], aiming at localizing and quantifying single- and multi-damage scenarios in a finite element model of a simply supported beam under simulated moving loads on bridges. SGF is a noise-reduction filter used in signal processing that approximates the underlying trend in the time-series data for smoothening polluted signals. Unlike the previously discussed techniques that exclude the baseline from the damage identification process, the study suggested estimating the baseline using the Gaussian curve fitting method, assuming that the baseline data points would conform to a normal distribution. However, the effectiveness of the proposed technique can be significantly compromised in in-situ applications, where the true baseline of complex bridges cannot be estimated with certainty, and damage-induced disturbances in their measured responses might be unintentionally removed during the smoothening process.

In summary, although the reviewed literature underscores the significant potential of time domain-based techniques for real-world bridge applications, an effective decision-making process regarding maintenance, repair, and rehabilitation of bridges requires that different types of damage be distinguished and the actual, not the estimated, severity of the damage be accurately quantified. A comprehensive BSHM system incorporating such reference-free techniques can contribute to prediction of the remaining service life of bridges.

## 5. Reference-Free Space Domain-Based Techniques

Localized structural damage, such as cracks, exerts discernible effects on the dynamic response within the space domain where the damage exists. This section delves into the space domain-based techniques that primarily focus on analyzing the dynamic response for detecting damage-induced effects without relying on a reference state for comparison. In this context, the space domain response encompasses various structural characteristics, including mode shapes, curvature profiles of mode shapes, operation deflection shapes, and their derivatives, covering either specific regions or the entire span of a bridge. Traditionally, these responses are derived indirectly through analysis of measurements taken in the time domain [2]. However, recent advancements in measurement equipment, such as scanning laser Doppler vibrometers [57,58,59] and high-resolution video cameras [60], now facilitate direct acquisition of the response in the space domain. Reference-free techniques that leverage space domain responses, whether obtained directly or indirectly, can be categorized into the approaches discussed below for structural damage detection.

### 5.1. Variations in Reference-Free Space Domain-Based Techniques

**Figure 5:**
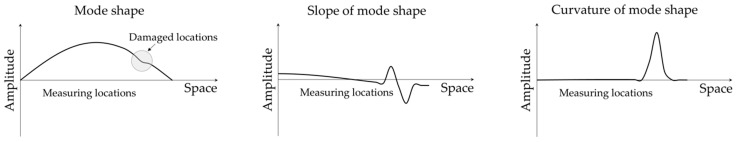
Typical damage-induced effects in the mode shape and its derivatives.

Generally, the space domain-based techniques are based on the premise that intact structures (i.e., at the reference state) exhibit smooth mode shapes, while damages introduce abrupt shifts in the slope of mode shapes or sudden peaks in curvature near the damaged locations [61]. Figure 5 illustrates an example of such damage-induced effects in a mode shape and its derivatives. Given that baseline mode shape data for structures in their reference state are typically unavailable, researchers often substitute them with smoothed versions of mode shapes derived from the damaged structures (i.e., the current state). Most space domain-based investigations have focused on assessing the efficacy of diverse curve-fitting methods for obtaining the smoothed mode shape curves, which serve as baselines for comparison with mode shape data points extracted from the damaged structures. This comparison facilitates precise localization of the damage.

**Figure 6:**
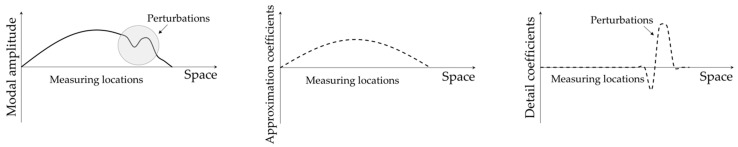
Detection of damage-induced perturbations in a modal response using wavelet coefficients.

In an alternative approach, some researchers conceptualize damage effects as noises—perturbations and irregularities—emanating in the space domain responses, attributed to their association with high-frequency events. Unlike global environmental and measurement noises, damage-induced noises impact responses solely at the damaged locations. Additionally, damage-induced perturbations can influence the dynamic equilibrium equations of structural components at damaged locations due to damage-related discontinuities and non-linearities in material and geometric properties [62]. Through a comprehensive signal processing and statistical analyses, Voggu and Sasmal [62], have demonstrated that even in the presence of inherent material nonlinearity in structural systems, damage-induced nonlinearity in the dynamic equilibrium equations prevails and can be identified even in early stages of damage, such as the initiation of breathing cracks in reinforced concrete bridges. Consequently, the detection of perturbations within space domain responses or dynamic equilibrium equations has been a focal point for a group of researchers [63,64,65,66,67]. Although WT is typically used for analyzing responses in the time domain, this group of researchers adapted it to analyze responses in the space domain for detecting damage-induced perturbations [68]. This adaptation involves substituting the time variable in the WT mathematical equations with the spatial variable. As illustrated in Figure 6, the approximation coefficients of the WT can represent the smooth component of the modal response (i.e., the baseline), while the detail coefficients reveal the damage-induced perturbations. Additionally, WT and other signal filtering tools can be employed to extract damage-induced perturbations from responses contaminated by other types of noise.

Apart from the diverse approaches to damage detection, the space domain-based techniques reported in the literature differ in their experimental implementation. The type of structure, damage, source of dynamic excitation source, and response measurement, etc., are some of the key parameters influencing applicability of the techniques. The subsequent subsections provide a concise overview of published work on space domain-based techniques, encompassing both curve-fitting and WT methodologies.

### 5.2. Review of Space Domain-Based Techniques Using the Curve-Fitting Approach

In 1996, Stubbs and Kim [22] conducted a pioneering study on a space domain-based technique that dispensed with the need for any data from a reference undamaged state. Their technique could localize damage and gauge its severity using only mode shape data from the damaged structure. However, they estimated the baseline data through a system identification method to obtain the modal parameters of the structure in its undamaged state. The baseline data were estimated in two steps: (1) Generating a generic finite element (FE) model to estimate the structure’s reference state; and (2) Calibrating the FE model with experimental data from the current state. By comparing the curvatures of mode shapes from the current state with those derived from the estimated reference state, they could identify damage and estimate its severity. The damage severity was represented by changes in the structural element stiffness.

To validate their approach, Stubbs and Kim experimentally located and quantified an imposed damage in a 4.9 m long two-span aluminum beam with a box-shaped cross section. The imposed damage consisted of a crack in the web near mid-span. The curvature of the modified experimental mode shape, which represented the damaged state of the beam, was then calculated, and compared with the curvature of the numerical mode shape, which represented the reference undamaged state of the beam. Although the technique successfully localized the imposed damage, it tended to overestimate its severity.

While the technique showed feasibility of the space domain-based damage identification in beam-like structures using data only from their current state, its experimental implementation was confined to detection of one type of damage in continuous beams only. Moreover, the technique relied on high-resolution spatial measurements, which could pose challenges in practical applications due to spatial sensitivity constraints [69]. The higher the resolution of the spatial measurements, the more mode shape data points can be obtained. Otherwise, some sort of interpolation will be needed to estimate the complete response of the structure. However, reliance on interpolation of data points between measurement locations may increase the risk of missing a damage located between measuring points. Furthermore, the overuse of estimation and simplification in the FE modeling for predicting mode shapes of the structures in their reference state may considerably reduce reliability of the technique. An inaccurate FE model can significantly affect the results of the damage detection process [21].

Ratcliffe [70] proposed a space domain-based technique that eliminated FE modeling from the damage detection process and operated solely on the mode shapes of the damaged structure. The technique was based on the premise that the undamaged structures exhibit smooth mode shapes, while damage induces abrupt changes in the slope of the mode shape in the vicinity of the damaged locations. Since measured mode shape data were discrete in space, Ratcliffe used a finite difference approximation based on the Laplacian operator to detect and quantify the sudden changes in the slope. A Laplacian operator was applied to the discretely measured mode shape data points to obtain the complete mode shape response of the damaged structure, while the mode shape in the reference state of the structure was estimated by fitting a smooth cubic polynomial. As an example, the Laplacian element, ${ℒ}_{i}$, corresponding to the $i^{th}$ discrete mode shape data point at spatial position $x_{i}$ and amplitude of $y_{i}$ is defined by Equation (5). Also, the cubic polynomial function for the Laplacian element, ${ℒ}_{i}$, can be expressed as $a_{0}+a_{1}x_{i}+a_{2}x_{i}^{2}+a_{3}x_{i}^{3}$, in which the coefficients $a_{0}$, $a_{1}$, $a_{2}$, and $a_{3}$ can be determined using Laplacian elements ${ℒ}_{i-2}$, ${ℒ}_{i-1}$, ${ℒ}_{i+1}$, and ${ℒ}_{i+2}$. The location of the targeted damage can be determined from the difference, $\delta_{i}$, between the cubic polynomial function and the Laplacian element ${ℒ}_{i}$, as expressed by Equation (6) [70].

(5) $${ℒ}_{i}=y_{i+1}+y_{i-1}-2y_{i},$$

(6) $$\delta_{i}=a_{0}+a_{1}x_{i}+a_{2}x_{i}^{2}+a_{3}x_{i}^{3}-{ℒ}_{i}.$$

**Figure 7:**
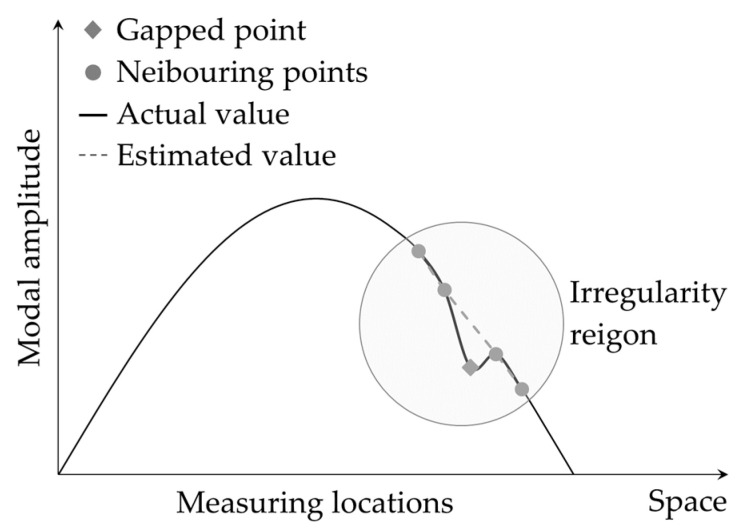
A schematic of the GSM [70] for quantification of damage-induced irregularities.

This procedure was later referred to as the *gapped smoothing method* (GSM) for damage detection [71]. The GSM is in fact a local curve-fitting method that utilizes a cubic polynomial function with neighboring four data points from a gapped middle point. Figure 7 depicts the basic idea of the GSM that the value of a data point on a smooth modal curve (i.e., the baseline) can be accurately estimated by the neighboring data points unless the point is in a region with damage-induced irregularities. Therefore, the difference between the actual value of the data point and its estimated value can be used to quantify the damage-induced irregularities in the curve.

Ratcliffe’s proposed technique was validated through a limited experiment on a small flat steel beam suspended with rubber cords at its two ends to represent free-free boundary conditions. A slot cut through the beam thickness at mid-length and across half the width induced damage, which the technique successfully located.

The concept of detecting damage by identifying sudden changes in modal parameters, such as mode shapes and strain energy, inspired several researchers to develop similar reference-free DITs employing the curve-fitting approach. Randhawa and Bhalla [72] slightly expanded the technique proposed by Ratcliffe [70] to detect multiple damage in beam-like structures. Yang et al. [73] incorporated the GSM into a strain energy-based technique to identify both the location and severity of single or multiple damage in beam-like structures. Ratcliffe and Bagaria [71] successfully employed the GSM to locate a manufactured delamination in a small-scale glass-reinforced epoxy beam. The GSM approach was extended to two-dimensional structures, such as plates [21,74,75,76], using a cubic polynomial with two variables to represent curvature of the plate. Subtracting the fitted curvature of the undamaged state from the calculated curvature of the damaged state of plates resulted in peaks at the damaged locations.

Wu and Law [21] conducted numerical simulations to assess the two-dimensional GSM’s effectiveness under various support conditions, measurement noise and resolution, and mode truncation. In a more comprehensive study, Yoon et al. [74] validated the two-dimensional GSM method using a FE model of a plate, performed experiments on composite plates with induced delamination, and demonstrated the method’s implementation in a large composite hull structure affected by a manufacturing defect. In all cases, damage was localized without the need for either mathematical model or baseline data.

Jiao et al. [77] proposed a two-step damage detection technique using Chebyshev polynomial fitting. The Chebyshev polynomials are a popular sequence of orthogonal polynomials that help with minimizing the perpendicular distances of the data points from the fitted curve (i.e., the least squares) when the equations become excessively complicated and difficult to solve for higher-order curves. In the first step of the technique proposed by Jiao et al., the modal curvature of a damaged bridge was calculated through a central difference approximation conducted on the mode shape data points. In the second step, the modal curvature of the bridge reference state was estimated by six order Chebyshev polynomial fitting to the calculated modal curvature of the damaged state. The targeted damage could then be detected by comparing the two modal curvatures corresponding to the damaged and the reference states. Only a numerical simulation of a simply supported beam-type bridge was used to demonstrate application of the technique to damage localization.

A similar approach was taken in a study by Rucevskis et al. [20] aiming at detecting and localizing damage in plate-like structures. After obtaining the mode shape curvature data of the damaged plate, a regression analysis with a polynomial approximation was employed to estimate the smooth mode shape curvature surface of the undamaged plate. A damage index was defined as the absolute difference between the measured curvature of the damaged plate and the estimated curvature of the undamaged plate, where the maximum value of the index could be an indication of damage. The technique was examined through several sets of numerical simulations considering different levels of damage severity as well as measurement noise and resolution. The technique’s practicality was investigated experimentally in an aluminum plate containing mill-cut damage. It was found that a major drawback of the technique was that the severity of the damage needs to be relatively high for successful detection. Additionally, the high-frequency dynamic excitation required in this technique significantly limits its practical applications in in-situ cases.

**Figure 8:**
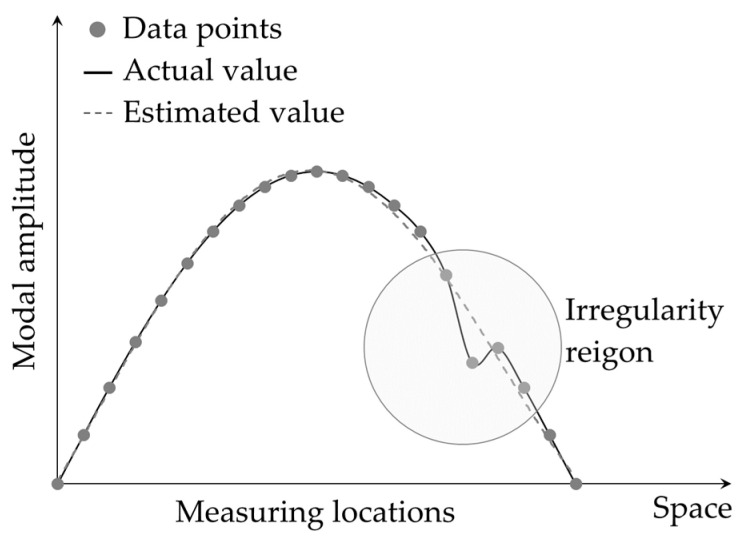
A schematic representation of the GFM [79] as a global curve-fitting method.

Despite the successful applications of GSM-based and similar damage detection techniques [78], certain challenges, such as the smearing effect on the edges of damage and fault detections near the boundary edges, have prompted researchers to seek improvements. In response, Yoon et al. [79] introduced the global fitting method (GFM) as a means of enhancing damage localization in beam-like structures, building upon previously developed methods. While the GSM technique involves local curve fitting (refer to Figure 7) of the mode shape curvature, the GFM is a global curve fitting (refer to Figure 8) for the entire mode shape. The GFM is particularly interesting for representing the baseline mode shape data because mode shapes generally reflect the global dynamic behavior of structures. Therefore, calculating curvature of the mode shape obtained by the GFM provides a more accurate estimation of the reference state compared to that derived from the GSM. Improved quality of baseline data can reduce errors and enhance the precision of damage detection. In essence, the GFM utilizes a generic form of mode shape, expressed in Equation (7), to fit a smooth curve to the all the mode shape data points.

(7) y(x)=Acos(λx)+Bsin(λx)+Ccosh(λx)+Dsinh(λx),

where x and y(x) are the position and the amplitude of mode shape, respectively, λ is determined through solution of the characteristic equation governing beam vibration, and the coefficients A, B, C, and D can be calculated by applying the beam boundary conditions and employing a curve-fitting method such as the Gauss–Newton method. In situations where a structure, such as a bridge, is complex or lacks predefined boundary conditions, superposition of predefined mode shape forms can be employed [79].

Performance of the GFM-based damage detection technique was also evaluated by Yoon et al. [79] through FE analysis and experimental data obtained from two flat steel beam specimens intentionally damaged by milling notches with varying severity. The technique proved capable of locating severe damage using both FE analysis data and experimental data. However, the GFM-based technique was able to successfully identify damage of slight severity only when using FE analysis data. The diminished performance of the GFM-based technique in detecting slight damage when using experimental data was attributed to noise in those data. This limited sensitivity to detecting slight damage presents challenges for the practical application of the technique in in-situ scenarios.

To improve damage detection in noisy conditions, He and Zhou [80] proposed a technique to enhance the reconstruction of baseline mode shapes from data measured at the structures damaged state. As an added interim step in the damage detection process, the proposed technique performed a statistical analysis of the measured data to remove undesirable effects of external sources of vibration, such as measurement and/or environmental noises, from the structure’s natural mode shapes. However, even this technique required modal analysis to obtain the natural frequencies and mode shapes from the measured data [81]. Removing this requirement from the damage detection process is of great importance as the modal analysis is an extra data processing effort subject to engineering judgement [69]. Also, the obtained modal data that present the structural behavior at resonance are often less sensitive to damage than the data obtained at frequencies away from resonance [82]. Furthermore, the modal analysis uses a limited set of measured data—only the ones related to the resonance frequencies—while a higher sensitivity to damage can be achieved if all data sets could be utilized in the damage detection process [69].

**Figure 9:**
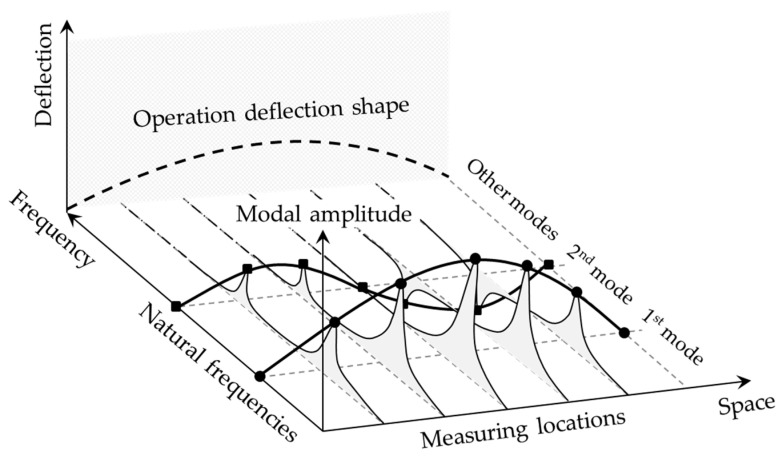
A schematic comparison of ODS [81] and mode shapes.

Recognizing these limitations, Gao et al. [75,76] proposed a non-modal damage localization technique utilizing frequency response functions (FRF) [83] combined with a two-dimensional GSM to detect and localize damage in plate-like structures. Unlike mode shapes and their derivatives that were based on natural frequencies, the FRF shapes and their derivatives were based on a wide range of frequencies; hence, provided more information on the location of damage. Furthermore, GSM- and GFM-based techniques were employed on the operation deflection shape (ODS) of the structures instead of their mode shapes [69,81,84]. As shown in Figure 9, while mode shapes represent the deformation response of a structure at its natural frequencies, the ODS consists of the deformation responses at every frequency component involved in the dynamic excitation. Unlike the mode shapes, which are inherent properties of bridge structures, the ODS depends on the operational condition of bridges.

The robustness of using the ODS in space domain-based damage detection was investigated by many researchers. Experimental implementation of a GSM-based damage detection technique in small-scale lab specimens [69] and large-scale structures [84] showed that the use of the ODS instead of mode shapes could significantly improve the technique’s sensitivity to damage with slight severity. Yoon et al. [81] investigated a GFM-based technique in identification of the location and extent of notches in steel beams, delamination in composite beams and plates, and dry spots in a composite hull structure. The obtained results showed enhanced performance of the technique when the ODS data were used rather than mode-shape data. Advanced methodologies, such as the use of continuously scanning laser doppler vibrometer systems, for obtaining the ODS and its derivatives for beam- and plate-like structures can be found in [57,58]. Cao and Ouyang [85] studied the effects of noises and sources of uncertainties on the robustness of damage localization techniques using the ODS. A comparative study was conducted by Zhang et al. [86] on the performance of the GSM- and GFM-based damage detection techniques using the ODS. Both numerical simulation and experimental implementation in beam- and plate-like structures verified superiority of the GFM-based technique using the ODS.

**Figure 10:**
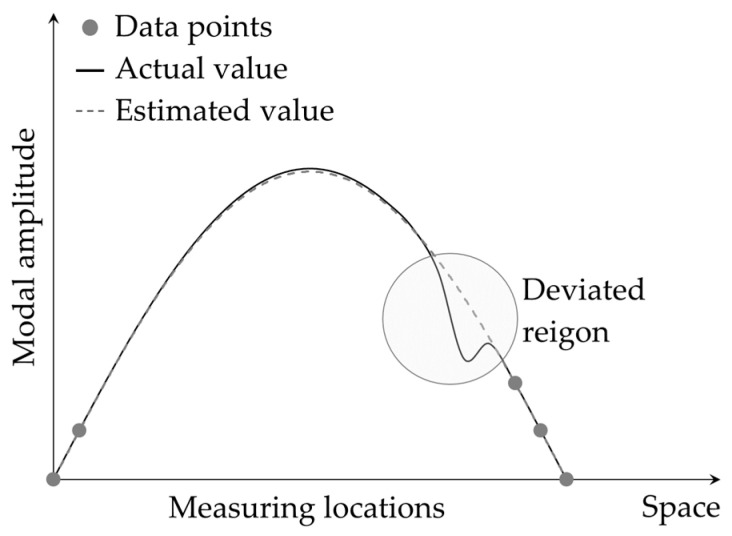
A schematic of the GDM [87] as a global curve-fitting method.

Kim et al. [87] introduced a technique similar to the GFM that followed the global approach in curve fitting. The technique was called the *global-deviation method* (GDM) and was able to detect damage by identifying the mode shape data points that abruptly deviated from a globally estimated mode shape curve through calculation of a damage index (DI) defined by Equation (8).

(8) $$DI=y_{i}-\left[A\cos(\lambda x)+B\sin(\lambda x)+C\cosh(\lambda x)+D\sinh(\lambda x)\right],$$

where $x_{i}$ and $y_{i}$ are respectively the position and the amplitude of $i^{th}$ measured mode shape data point. The parameters λ, A, B, C, and D can be determined using the five data points near the two ends of the measured mode shape and are used to estimate the smooth mode shape curve representing the baseline (see Figure 10). The effectiveness of the GDM-based technique was illustrated through a numerical simulation and an experimental implementation in a cantilever beam. However, the technique assumed that the locations near the two ends of the beam were undamaged and neglected the difference in the natural frequencies of the damaged and undamaged states of the beam. Such assumptions may not hold in practice, making the technique less suitable for practical applications.

A study on the GDM-based technique conducted by Lee et al. [88] showed that the use of strain gauges is more practical than the use of accelerometers for data measurement near the supports of a beam for the purpose of establishing the baseline mode shape curve of the beam. To reduce sensitivity of the GDM-based technique to experimental noises, Lee and Eun [89] proposed a technique using an additional mass with changing position for intentionally altering the physical properties of a damaged beam. The additional mass was moved from one measuring location to another, and the response of the beam was measured for each position under dynamic excitations applied with an impact hammer. Comparing the obtained displacement responses with each other could determine the damaged location. The inherent assumption in this technique was that the additional mass could replace the stiffness reduction due to damage. Hence, when the additional mass was positioned in the vicinity of the damaged location, the abrupt change in the displacement response could locate the damage. However, in reality, structural damage is significantly more complicated than this simplified assumption. Also, it may not be feasible or practical to move an additional mass along all the measuring locations on a large structure.

While most of the research studies discussed in this section focused on the detection and localization of damage using different curve-fitting approaches on the modal or non-modal data, not much effort was made to quantify or even estimate the size, extent, and severity of damage. He [90] and He et al. [91,92] presented a two-step technique for quantitative characterization of delamination in composite structures and thickness reduction in metallic structures. In the first step of the technique, the GSM was employed to determine the location of damage from curvature data. In the second step, the severity of the located damage was estimated by iteratively matching the responses obtained from a FE model and the actual structure. Numerical and experimental validations of the technique were investigated using a scanning laser vibrometer. The damage and its extent could be correctly identified when the potential damaged locations were limited. In addition, the performance of the technique was affected in cases with complex forms of delamination, such as that caused by impact. It was also found that the vibration responses were highly sensitive to boundary conditions. This imposed significant limitations on the effectiveness of the second step of the technique.

In summary, the space domain-based techniques discussed in this section employed various curve-fitting methods on modal and non-modal responses of structures to detect and localize damage, and sometimes estimate its severity. While these techniques have evolved theoretically over the years, their practical implementation in full-scale structures remains rare or unapparent.

### 5.3. Review of Space Domain-Based Techniques Using Wavelet Transform (WT) Approach

Much like time domain responses, space domain responses experience damage-induced perturbations in the vicinity of the damaged locations. While these localized perturbations might not be readily apparent in the space-domain responses, they can often be detected through the application of WT. The underlying principle is that the presence of perturbations in the response can be identified by scaling the basic wavelet function, and the location of these perturbations can be determined by shifting the function across the entire response. Numerous studies have explored the application of WT to identify structural damage by analyzing dynamic responses measured in the space domain. Comprehensive reviews of these studies can be found in [14,63,93,94,95,96]. However, many of these techniques are reference-based, implying that the wavelet coefficients corresponding to the structural response in the undamaged reference state are typically needed as baseline data. As discussed earlier in this paper, the efficacy of reference-based techniques often encounters significant limitations in most practical in-situ applications. Therefore, recent efforts have focused on the development of reference-free space domain-based DITs using the WT approach. A review of these studies is presented below.

Zhong and Oyadiji [63] introduced a crack detection technique for beam-like structures utilizing the stationary wavelet transform (SWT) to the mode shapes. The concept rests on the assumption that the mode shape of a cracked beam resembles that of an uncracked beam but with localized perturbations induced by the crack. Decomposition of the mode shape of the cracked beam through SWT yields approximation coefficients depicting a smooth curve of the uncracked beam and detail coefficients revealing the crack-induced perturbations. The differences between the detail coefficients of the two halves of the beam provide information on the presence and location of the cracks. Experimental validation on FE models of a series of simply supported cracked beams showcased efficacy of the technique under varying crack location, size, and depth, as well as the measurement resolution. The mode shapes were intentionally contaminated with random noise to simulate real-world conditions. The technique demonstrated capability in detecting cracks without the need for mode shapes from the uncracked state. However, because one half of the beam served as the reference for the other half, data from the reference half might also be influenced by the crack, potentially leading to false-negative damage indications. Moreover, this technique was only applicable to symmetrical and anti-symmetrical beam-like structures.

Zhong and Oyadiji [64] extended their study through numerical and experimental components, testing the technique on a 2.4 m simply supported aluminum beam containing a single crack. Zhou and Li [97] employed two-dimensional WT to detect damage in plate-like structures, focusing on identifying damage in a single lattice truss core of a small-scale composite sandwich panel. Dynamic excitations using a shaker were applied to the beam and the plate tested respectively in [64] and [97], and mode shapes were acquired accordingly. The tests showed that accuracy of the technique was proportional to the accuracy of the mode shape measurement. Therefore, high-resolution measurements were required. This requirement poses challenges when applied to real-world structures. This is a common issue with space domain-based techniques, particularly for the ones applying WT to the space-domain response of structures. The authors recommended the use of scanning laser vibrometers to achieve high resolution measurements. Nevertheless, the use of shakers or laser vibrometers to induce and measure dynamic excitations might encounter challenges in large-scale structures such as bridges. Recently, digital video cameras have been used to provide high spatial measurement resolution. Combined with advanced image processing tools, the use of video cameras can be a promising solution for high-resolution dynamic measurements of operating bridges. A feasibility study on damage detection using high spatial resolution mode shapes extracted from captured videos of operating structure is reported in [60]. Advances in wireless communication, robotics, and video capturing systems have shown promise for achieving high-resolution extraction of mode shape data from dynamic response of bridges [28,98]. Through these advancements, a limited number of mobile sensors can be sufficient to perform high spatial resolution measurements. However, most space domain-based techniques presently compatibility with data obtained from mobile sensors. Therefore, development of mode shape extraction techniques that can be integrated with mobile sensors data has become the focus of some studies [99,100,101].

Xu et al. [65] developed a technique using dynamic equilibrium equations to detect multiple cracks in beam-like structures. The technique locally examined the dynamic equilibrium of structural components and could detect perturbations in the equilibrium equations resulting from damage-induced discontinuities in material and geometric properties at the boundaries of the damaged locations. However, practical implementation was hindered by the potential significant confusion of noise-induced perturbations with the damage-induced perturbations. Consequently, Xu et al. [64] enhanced the technique’s accuracy by employing WT as a filter to distinguish between the two types of perturbation and isolate the damage-induced effects from the responses contaminated by noise. Experimental validation was conducted to assess the technique’s effectiveness in identifying multiple cracks in a small-scale cantilever beam. Dynamic excitations were applied using an electromechanical shaker with a frequency bandwidth of 2000 Hz, and response data in the space domain were collected using a scanning doppler laser vibrometer.

The concept of extracting damage-induced perturbations from the dynamic equations and irregularities from the mode shapes using WT, or other filtering methods [67], garnered interest from other researchers. The dynamic equilibrium-based technique was later expanded for application in plate-like structures [102,103]. In a similar wavelet-aided research, Bai et al. [23] conducted numerical and experimental examinations to identify delamination in composite plates under noisy conditions, showcasing improvements in the accuracy of damage detection in both beam-like and plate-like structures due to the use of WT in the response analysis. However, as previously discussed in this paper, it is important to note that this type of technique may not be readily applicable to in-situ cases due to significant differences in dynamic excitations and response measurement between the laboratory and the real-world conditions.

## 6. Summary and Guideline on the Use of Reference-Free Time and Space Domain Techniques

Table 2 provides a summary of the merits and shortcomings of various reference-free damage identification vibration-based techniques currently reported in the literature. Generally, these techniques are easy to use as they do not require intensive computations. However, they are capable only of detecting the presence of damage, determining its location, and in some cases estimating its severity without quantification. Therefore, they can be valuable in a preliminary stage of damage identification. The information in Table 2 should provide a guideline on the use of available time and space domain techniques for structural damage identification.

## 7. Summary and Conclusions

Structural assessment of bridges is indispensable in addressing global concerns regarding the safety and serviceability of bridge infrastructure. Utilization of BSHM systems for structural damage identification holds immense promise. Among the various techniques available for BSHM, vibration-based DITs have been one of the most suitable techniques for on-site applications. Traditional vibration based DITs involve comparison of the bridge response measured at its current (damaged) state with that obtained at its reference (undamaged) state. However, the efficacy of the reference-based damage identification process is significantly hampered by the unavailability of response data at the reference state and the considerable influence of varying operational and environmental conditions on the process. Consequently, the development of reference-free damage identification techniques becomes imperative. Reference-free BSHM techniques operate on the premise that the damage identification process can be achieved solely through the bridge response in its current state. This paper has comprehensively reviewed the state-of-the-art reference-free techniques employing responses obtained in either the time or space domain.

Time domain-based techniques typically employ advanced signal processing tools to analyze dynamic responses directly measured in the time domain, aiming to detect damage-induced effects. Statistical and entropy analyses are subsequently employed to generate damage indices for identifying the locations of damage and estimating its severity. Time domain-based techniques are generally more practical, and the existing literature highlights their potential applicability in large-scale structures.

Space domain-based techniques have garnered substantial attention as they are straightforward and easy to understand. However, their accuracy is heavily contingent on the resolution of the dynamic measurements in the space domain, rendering them challenging to implement in operational bridges. Recent advancements in measurement devices and sensors, combined with modern vision processing tools, hold promise for enabling high-resolution dynamic measurements of bridge responses. Therefore, more research studies are expected to focus on vision-based DITs relying exclusively on responses obtained from the current state of bridges.

A key limitation of most reference-free DITs is their reliance on shock and vibration testing shakers, primarily used for exciting small-scale laboratory specimens across a broad frequency range. In contrast, ambient vibrations, which typically excite bridges within a narrow band of low frequencies, serve as the only practical source of dynamic excitation in most in-situ scenarios.

Furthermore, while the literature reports reference-free DITs primarily achieving damage detection and localization in small-scale structures, successful damage severity estimation in large-scale structures remains infrequent. Consequently, a compelling need persists for further research studies aimed at surmounting the practical obstacles that impede implementation of reference-free vibration-based DITs into BSHM systems. Addressing these challenges will undoubtedly contribute to enhancing the practical viability of reference-free techniques in real world applications.

## 8. Recommendation for Further Research

Prediction of the remaining service life of bridges is a pivotal objective of BSHM systems. To achieve this objective, characterizing the various types of damage and quantifying the actual damage severity with a high degree of certainty are essential. However, currently available reference-free techniques, whether time or space domain-based, have not demonstrated the ability to characterize different types of damage and quantify actual, as opposed to estimated, damage severity. Therefore, further enhancements in the effectiveness of reference-free techniques are essential. To achieve this objective, it is recommended to integrate a Finite Element (FE) model updating procedure with a time domain-based DIT, such as the wavelet entropy (WE)-based technique.

In conventional FE model updating, the objective is to minimize the difference in the modal properties (e.g., natural frequencies and mode shapes) between the target structure and its numerical model. Minimizing this difference is achieved by continuously updating the FE model through adjusting the structural properties of its elements and their boundary conditions, until it eventually approximates the measured modal properties of the target structure, thereby serving as a representative model capable of identifying damage locations and quantifying its severity in terms of losses in element stiffness and/or undesired changes in connections.

However, modal properties often reflect the global dynamic characteristics of structures, while damage is typically a localized phenomenon. Moreover, modal properties are sensitive to variations in operational and environmental conditions, which potentially obscure damage effects on the dynamic response. Therefore, adjusting the FE model based on measured modal properties can be highly ineffective for damage identification and quantification.

In the hybrid FE-WE technique proposed herein, an objective function is defined for each measuring location as the difference in the WE index between the target structure and its numerical model. A linear combination of all the location-based objective functions leads to a global objective function (*GOF*) expressed as:

(9) $$GOF=\sum_{i=1}^{m}w_{i}\left|WE_{i}^{model}-WE_{i}^{structure}\right|,$$

where m is the total number of measuring locations in the structure; $w_{i}$ is a weighting factor for the objective function at location i; $WE_{i}^{structure}$ and $WE_{i}^{model}$ are the WE index at location i of the target structure and its FE model, respectively. Optimizing the *GOF* expressed by Equation (9) involves updating the FE model and recalculating the WE indices for the updated model.

In the context of FE model updating for bridges, *GOF* formulation as a linear combination of individual objective functions has been effectively employed in some studies [73,104,105], in which functions related to natural frequencies, mode shapes, and static deflections were used. A common way to ensure convergence and accuracy of the *GOF* optimization was to allocate a weighting factor to each individual objective function. The weighting factor can adjust the contribution of the individual objective functions to the *GOF* based on several factors such as reliability of the measured data, confidence in the FE modeling of specific components, significance of an individual objective function relative to others, etc. [73,104,105,106,107,108]. Therefore, the allocation of weighting factors is often case-specific and determined through evaluation and decision-making processes [109]. In general, when effective devices and sensors are used for measuring the structural response, and robust techniques are adopted for processing the measured data and mitigating the undesirable effects of varying operational and environmental conditions, and provided detailed well-correlated FE models of the structure are available for damage identification, the most significant individual objective function is allocated the highest weighting factor. Weights can be set directly or by employing multi-attribute decision-making techniques such as Simple Additive Weighting (SAW) [110], Analytic Hierarchy Process (AHP) [111], Step-Wise Weight Assessment Ratio Analysis (SWARA), or some simple but effective weight assigning methods such as Rank Order Centroid Method, Ratio Method, Rank Sum Method, etc. Some of these techniques are reviewed and evaluated by Vinogradova [109]. Despite the variety of available weight assigning techniques, there is no consensus among researchers on the best or most appropriate technique. However, the agreement is that the sum of all the weights should be equal to 1.

An alternative approach that can be adopted to mitigate the difficulty associated with weighting combined objective functions is to employ evolutionary multi-objective optimization techniques [112] such as Genetic Algorithm (GA) [113,114] and Particle Swarm Optimization (PSO) [115]. For application in structural damage identification, Perera et al. [116] developed an evolutionary GA and PSO multi-objective framework with objective functions based on modal flexibilities, frequencies, and mode shapes.

For the hybrid FE-WE technique proposed herein, the WE indices can be obtained through the steps discussed in Section 4.1, utilizing any of the techniques reviewed in Section 4.2. For example, using the wavelet entropy technique discussed in [48,49,50,51,52,53,54,55], a relative WE index at a location ℓ is calculated as:

(10) $$WE_{\ell }=\frac{1}{m-1}\sum_{i=1}^{m}\sum_{j=1}^{n}p_{ij}\;\ell n\left(\frac{p_{ij}}{p_{\ell j}}\right),$$

in which the wavelet energy, $\left\{P_{\ell }\right\}$, of the response obtained at location ℓ is compared against the wavelet energy of the responses obtained at m−1 other locations through n levels of wavelet decomposition; $p_{ij}$ is the wavelet energy corresponding to decomposition level j for the response obtained at location i.

Application of Equation (10) to the responses obtained from the target structure and the FE model results in ${WE}_{\ell }^{structure}$ and ${WE}_{\ell }^{model}$, respectively. The *GOF* can be obtained by repeating this process for all the measuring locations and substituting the resulting WE indices in Equation (9).

One advantage of employing wavelet entropy analysis over modal analysis is that the wavelet coefficients can provide valuable information on both the global and local dynamic characteristics of the structure. The approximation coefficients extracted from higher levels of wavelet decomposition can be used to adjust the global dynamic characteristics of the FE model, such as global stiffness and mass. This is because these coefficients capture information on the low-frequency components of the response, which are typically linked to the structure’s global vibration. On the other hand, the detail coefficients extracted from lower levels of wavelet decomposition can be used to adjust the local dynamic characteristics of the FE model, such as element stiffness and connectivity. This is because detail coefficients capture information on the high-frequency components, often associated with the structure’s local vibration.

This advantage allows for a gradual optimization process for the *GOF* to ensure a proper balance between accuracy and computational cost. The process commences with the calculation of the WE indices solely using the approximation coefficients extracted from higher levels of wavelet decomposition. This initial step can lead to quick convergence. However, at this stage, the FE model primarily represents the global dynamic characteristics of the target structure and is not sufficiently accurate for damage quantification, as damage is inherently local. Gradually, the detail coefficients from lower wavelet decomposition levels are incorporated into the WE index calculation, prompting updates to the FE model with more detailed and adjusted properties. Ultimately, this process yields an accurate FE model capable of representing not only the global but also the local dynamic characteristics of the target structure and its elements. This FE model can then be harnessed for reference-free damage identification and quantification in the target structure.

Another benefit of the hybrid FE-WE technique is its ability to mitigate the impact of varying operational and environmental conditions on the FE model adjustment and damage quantification. This is because the WE indices are calculated via a relative analysis that can eliminate the common effects of the varying conditions on the measuring locations [51]. Additionally, by defining an individual objective function for each measuring location, the hybrid FE-WE technique introduces multiple constraints to the *GOF*, thus reducing the number of feasible solutions and enhancing the performance of the optimization process. Furthermore, the FE model can assist in determining the optimal basic wavelet function, decomposition level, and dynamic response measurement arrangement [46].

## Figures and Tables

**Table 1:** Summary of inefficiencies imposed by reference state.

Process	Brief Description	Challenges and Shortcomings
Access	Process of obtaining data from intact state of structures	Inherent existence of damage in all engineered systems Lack of valid baseline data for in-service bridges, where they have already been exposed to damaging conditions Difficulty with obtaining dynamic properties of pristine structures in most in-situ cases
Interpretation	Process of identifying damage through detecting changes in obtained data	Unreliable reference state, where first set of measured data is assumed to be true representative of undamaged state False-positive damage indication, where changes due to varying conditions are wrongly interpreted as damage False-negative damage indication, where changes due to varying conditions mask changes induced by damage

**Table 2:** Summary of reference-free vibration-based DITs.

Domain	Damage Identification Strategy	Merits and Shortcomings
Time	Detecting disturbances using: - CWT [44,45] - DWT [40,48,49,50,51,52,53,54,55] - WPT [36,41,42,46,47]	Merits: - Direct use of measured responses - Capable of estimating damage severity - Demonstrated potential for implementation in large-scale and complex structures Shortcomings: - Applicable mostly to structures with identical elements - Difficult interpretation of signal processing outcomes
Space	Curve fitting using: - GSM [21,70,71,72,73,74,78] - GFM [97] - GDM [87,88,89] - Regression [20,77] - FRF [75,76,83] - ODS [69,81,84,86] - FE models [22,90,91,92] Detecting perturbations in: - Modal shapes [23,63,64,67,97] - Dynamic equations [65,66,102,103]	Merits: - Easy interpretation of outcomes as they express physical properties of structures - Demonstrated efficiency for damage detection and localization in small-scale structures - Availability of variety of extensively studied methods Shortcomings: - Require transformation of measured responses - Difficult interpretation of signal processing outcomes - Require prior knowledge of intact state for estimating damage severity - Require high spatial resolution of measurement - Susceptible to errors in response transformation and analysis, inaccurate curve fitting and FE modeling, etc.

## Data Availability

No new data were created for this review.
